# (*E*)-Benzyl 2-{4-[eth­yl(2-hy­droxy­eth­yl)amino]­benzyl­idene}hydrazinecarbodi­thio­ate

**DOI:** 10.1107/S2414314621009019

**Published:** 2021-09-03

**Authors:** Hui Guo, Wenli Du, Haoyu Zhou

**Affiliations:** a Department of Chemistry, Anhui University, Hefei, Anhui 230039, People’s Republic of China; University of Aberdeen, Scotland

**Keywords:** Schiff base, crystal structure, inter­molecular inter­actions

## Abstract

In the title compound, C_19_H_23_N_3_OS_2_, the dihedral angle between the aromatic rings is 86.80 (8)° and the tertiary amine grouping is almost planar (bond-angle sum at the N atom = 360.0°). In the crystal, pairwise N—H⋯O hydrogen bonds link the mol­ecules into inversion dimers, and O—H⋯S hydrogen bonds link the dimers into [101] chains.

## Abstract 2

In the title compound, mol­ecules are linked into chains by O—H⋯S and N—H⋯O hydrogen bonds.

## Graphical abstract

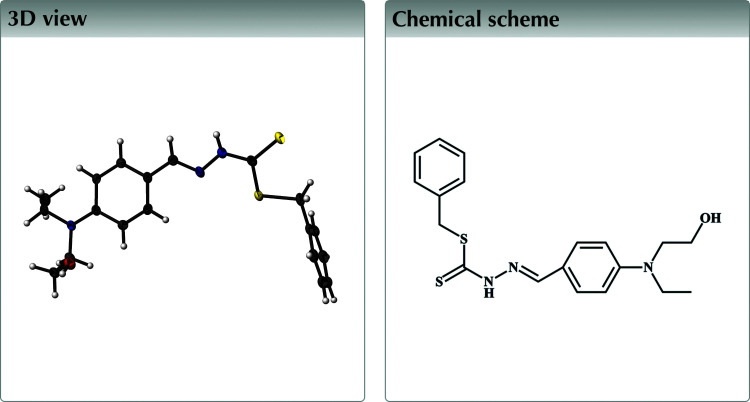

## Structure description

The title compound, C_19_H_23_N_3_OS_2_, is a *D*–π–*A* type Schiff base with an aniline derivative as the electron-donating (*D*) group and a hydrazino­thioic acid benzyl ester as the electron-withdrawing (*A*) group. Schiff base ligands based on benzyl hydrazino­thio­ate are an important class of compounds that have attracted widespread inter­est (Zhao *et al.*, 2008 ▸).

**Figure 1:**
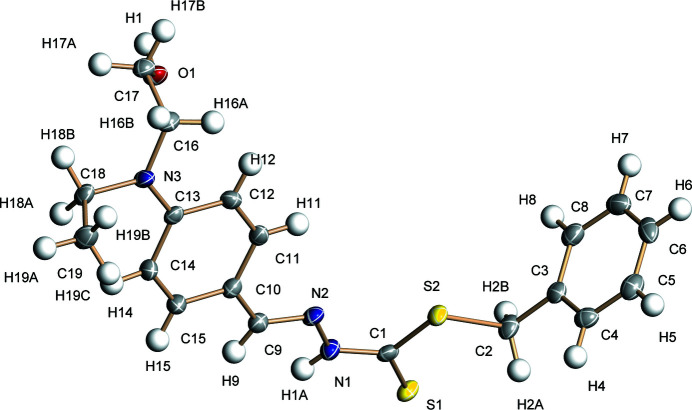
The mol­ecular structure of the title compound showing 50% displacement ellipsoids.

The crystal structure has triclinic (*P* 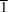 ) symmetry. The dihedral angle between the C3–C8 and C10–C15 benzene rings is 86.80 (8)° and the C1—N1—N2—C9 torsion angle is −170.6 (2)° (Fig. 1 ▸). This twisted conformation may effectively inhibit fluorescence quenching in the crystal by reducing π–π stacking between mol­ecules. The S1/S2/N1/N2/C1 grouping is close to planar (r.m.s. deviation = 0.026 Å) and the geometry at N3 is almost planar (bond-angle sum = 360.0°) and C17 and C19 point from C13/C16/C18/N3 in opposite directions [deviations = −1.411 (2) and 1.334 (2) Å, respectively].

**Figure 2:**
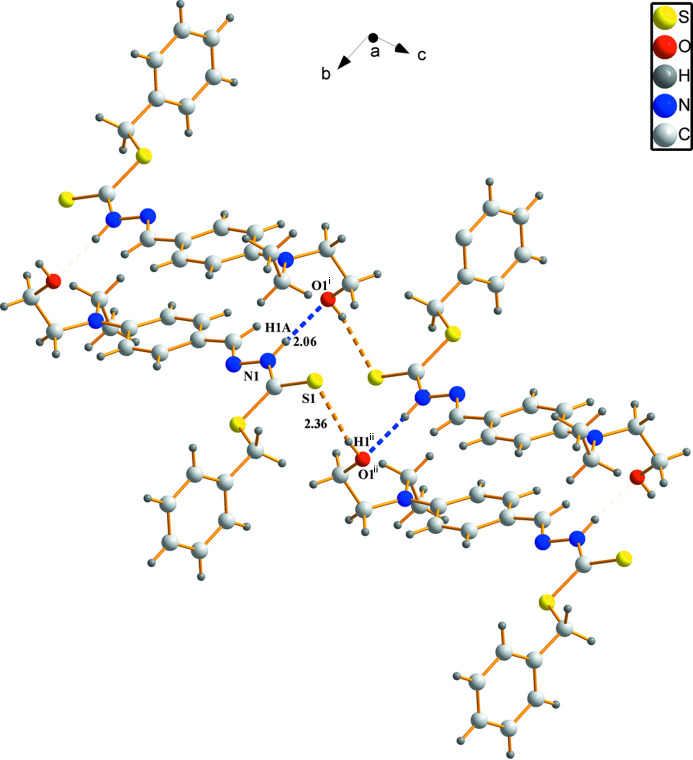
The inter­molecular hydrogen bond diagram of compound. Symmetry codes: (i) −*x* + 1, −*y*, −*z* + 1; (ii) *x* + 1, *y*, *z* + 1

In the extended structure, pairwise N—H⋯O hydrogen bonds (Table 1 ▸) generate inversion dimers featuring 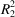 (22) loops, and O—H⋯S hydrogen bonds link the dimers into [101] chains (Fig. 2 ▸).

## Synthesis and crystallization

In a 100 ml round-bottomed flask, 3.40 g (0.17 mol) of benzyl­hydrazine carbon di­sulfide and 3.00 g (0.17 mol) of 4-(eth­yl(2-hy­droxy­eth­yl) amino) benzaldehyde were dissolved in 50 ml of ethanol and stirred at room temperature for 15 minutes and then transferred to an oil bath for reflux at 353 K for 3 h. After the reaction was cooled to room temperature, a yellow solid 5.10 g (yield 84%) was precipitated out and recovered by filtration. Colourless blocks were recrystallized from ethanol solution.

## Refinement

Crystal data, data collection and structure refinement details are summarized in Table 2 ▸.

## Supplementary Material

Crystal structure: contains datablock(s) I. DOI: 10.1107/S2414314621009019/hb4387sup1.cif

Structure factors: contains datablock(s) I. DOI: 10.1107/S2414314621009019/hb4387Isup2.hkl

Supporting information file. DOI: 10.1107/S2414314621009019/hb4387Isup3.cml

CCDC reference: 2106601

Additional supporting information:  crystallographic information; 3D view; checkCIF report

## Figures and Tables

**Table 1:** Hydrogen-bond geometry (Å, °)

*D*—H⋯*A*	*D*—H	H⋯*A*	*D*⋯*A*	*D*—H⋯*A*
N1—H1*A*⋯O1^i^	0.88	2.06	2.933 (3)	175
O1—H1⋯S1^ii^	0.84	2.36	3.1749 (19)	163

Symmetry codes: (i) 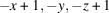 ; (ii) 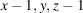 .

**Table 2:** Experimental details

Crystal data	
Chemical formula	C_19_H_23_N_3_OS_2_
*M*_r_	373.52
Crystal system, space group	Triclinic, *P* 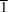
Temperature (K)	120
*a*, *b*, *c* (Å)	9.1794 (18), 9.4642 (19), 11.665 (2)
α, β, γ (°)	101.78 (3), 107.81 (3), 93.57 (3)
*V* (Å^3^)	936.1 (4)
*Z*	2
Radiation type	Mo *K*α
μ (mm^−1^)	0.30
Crystal size (mm)	0.12 × 0.11 × 0.1

Data collection	
Diffractometer	Stoe X-AREA CCD
Absorption correction	Multi-scan (*X-RED32*; Stoe, 2018 ▸)
*T*_min_, *T*_max_	0.342, 0.808
No. of measured, independent and observed [*I* > 2σ(*I*)] reflections	8565, 3419, 2508
*R*_int_	0.038
(sin θ/λ)_max_ (Å^−1^)	0.609

Refinement	
*R*[*F* ^2^ > 2σ(*F* ^2^)], *wR*(*F* ^2^), *S*	0.039, 0.097, 0.92
No. of reflections	3419
No. of parameters	228
H-atom treatment	H-atom parameters constrained
Δρ_max_, Δρ_min_ (e Å^−3^)	0.43, −0.25

Computer programs: *X-AREA* (Stoe, 2018 ▸), *SHELXT2018*/2 (Sheldrick, 2015*a* ▸), *SHELXL2018*/3 (Sheldrick, 2015*b* ▸) and *OLEX2* (Dolomanov *et al.*, 2009 ▸).

## full crystallographic data

### Crystal data

**Table:**

C_19_H_23_N_3_OS_2_	*Z* = 2
*M**_r_* = 373.52	*F*(000) = 396
Triclinic, *P*1	*D*_x_ = 1.325 Mg m^−^^3^
*a* = 9.1794 (18) Å	Mo *K*α radiation, λ = 0.71073 Å
*b* = 9.4642 (19) Å	Cell parameters from 8567 reflections
*c* = 11.665 (2) Å	θ = 69.8–4.3°
α = 101.78 (3)°	µ = 0.30 mm^−^^1^
β = 107.81 (3)°	*T* = 120 K
γ = 93.57 (3)°	Block, colourless
*V* = 936.1 (4) Å^3^	0.12 × 0.11 × 0.1 mm

### Data collection

**Table:**

Stoe X-Area CCD diffractometer	2508 reflections with *I* > 2σ(*I*)
φ and ω scans	*R*_int_ = 0.038
Absorption correction: multi-scan (X-Red32; Stoe, 2018)	θ_max_ = 25.6°, θ_min_ = 1.9°
*T*_min_ = 0.342, *T*_max_ = 0.808	*h* = −11→10
8565 measured reflections	*k* = −4→10
3419 independent reflections	*l* = −14→13

### Refinement

**Table:**

Refinement on *F*^2^	Primary atom site location: dual
Least-squares matrix: full	Hydrogen site location: inferred from neighbouring sites
*R*[*F*^2^ > 2σ(*F*^2^)] = 0.039	H-atom parameters constrained
*wR*(*F*^2^) = 0.097	*w* = 1/[σ^2^(*F*_o_^2^) + (0.0568*P*)^2^] where *P* = (*F*_o_^2^ + 2*F*_c_^2^)/3
*S* = 0.92	(Δ/σ)_max_ = 0.001
3419 reflections	Δρ_max_ = 0.43 e Å^−^^3^
228 parameters	Δρ_min_ = −0.25 e Å^−^^3^
0 restraints	

### Special details

**Table:**

Geometry. All esds (except the esd in the dihedral angle between two l.s. planes) are estimated using the full covariance matrix. The cell esds are taken into account individually in the estimation of esds in distances, angles and torsion angles; correlations between esds in cell parameters are only used when they are defined by crystal symmetry. An approximate (isotropic) treatment of cell esds is used for estimating esds involving l.s. planes.

### Fractional atomic coordinates and isotropic or equivalent isotropic displacement parameters (Å^2^)

**Table:**

	*x*	*y*	*z*	*U*_iso_*/*U*_eq_	
S1	1.15987 (6)	0.04410 (6)	0.90983 (5)	0.02333 (15)	
S2	1.05575 (6)	0.31931 (6)	0.83421 (5)	0.02330 (15)	
O1	0.22245 (18)	0.21535 (18)	0.18795 (14)	0.0262 (4)	
H1	0.223336	0.181675	0.115767	0.039*	
N1	0.8765 (2)	0.0761 (2)	0.79056 (16)	0.0233 (4)	
H1A	0.850789	−0.013576	0.794332	0.028*	
N2	0.7650 (2)	0.1512 (2)	0.73042 (16)	0.0228 (4)	
N3	0.1089 (2)	0.2823 (2)	0.39125 (16)	0.0224 (4)	
C1	1.0235 (2)	0.1382 (2)	0.84322 (19)	0.0223 (5)	
C2	1.2647 (3)	0.3598 (3)	0.9062 (2)	0.0290 (5)	
H2A	1.294123	0.355290	0.994245	0.035*	
H2B	1.315757	0.287256	0.863582	0.035*	
C3	1.3150 (2)	0.5093 (3)	0.8965 (2)	0.0238 (5)	
C4	1.3139 (3)	0.6313 (3)	0.9856 (2)	0.0273 (5)	
H4	1.276995	0.620316	1.051407	0.033*	
C5	1.3663 (3)	0.7686 (3)	0.9790 (2)	0.0308 (6)	
H5	1.365720	0.851217	1.040672	0.037*	
C6	1.4193 (3)	0.7867 (3)	0.8836 (2)	0.0320 (6)	
H6	1.455293	0.881218	0.879535	0.038*	
C7	1.4196 (3)	0.6659 (3)	0.7939 (2)	0.0335 (6)	
H7	1.455863	0.677709	0.728000	0.040*	
C8	1.3675 (3)	0.5285 (3)	0.7997 (2)	0.0298 (5)	
H8	1.367371	0.446318	0.737304	0.036*	
C9	0.6253 (2)	0.0890 (2)	0.6989 (2)	0.0228 (5)	
H9	0.606910	0.001568	0.723393	0.027*	
C10	0.4956 (2)	0.1479 (2)	0.6275 (2)	0.0220 (5)	
C11	0.5133 (3)	0.2530 (2)	0.5624 (2)	0.0231 (5)	
H11	0.614400	0.293822	0.571093	0.028*	
C12	0.3883 (2)	0.2988 (2)	0.4861 (2)	0.0231 (5)	
H12	0.405089	0.369235	0.442312	0.028*	
C13	0.2348 (2)	0.2430 (2)	0.47130 (19)	0.0208 (5)	
C14	0.2178 (3)	0.1427 (2)	0.5421 (2)	0.0240 (5)	
H14	0.117263	0.107096	0.539112	0.029*	
C15	0.3445 (3)	0.0952 (3)	0.6157 (2)	0.0255 (5)	
H15	0.328834	0.024729	0.659759	0.031*	
C16	0.1209 (3)	0.3845 (2)	0.3153 (2)	0.0243 (5)	
H16A	0.221483	0.447682	0.354456	0.029*	
H16B	0.038555	0.447687	0.313128	0.029*	
C17	0.1074 (3)	0.3110 (2)	0.1842 (2)	0.0243 (5)	
H17A	0.003408	0.255098	0.140883	0.029*	
H17B	0.122681	0.385034	0.138467	0.029*	
C18	−0.0480 (2)	0.2184 (3)	0.3739 (2)	0.0244 (5)	
H18A	−0.047229	0.114806	0.377521	0.029*	
H18B	−0.116034	0.221695	0.290540	0.029*	
C19	−0.1142 (3)	0.2960 (3)	0.4705 (2)	0.0324 (6)	
H19A	−0.216151	0.244927	0.457563	0.049*	
H19B	−0.123843	0.396399	0.462546	0.049*	
H19C	−0.045187	0.296706	0.553457	0.049*	

### Atomic displacement parameters (Å^2^)

**Table:**

	*U*^11^	*U*^22^	*U*^33^	*U*^12^	*U*^13^	*U*^23^
S1	0.0216 (3)	0.0248 (3)	0.0193 (3)	0.0069 (2)	0.0006 (2)	0.0039 (2)
S2	0.0180 (3)	0.0264 (3)	0.0225 (3)	0.0036 (2)	0.0004 (2)	0.0086 (2)
O1	0.0268 (9)	0.0339 (9)	0.0189 (8)	0.0075 (7)	0.0093 (7)	0.0048 (7)
N1	0.0204 (10)	0.0239 (10)	0.0225 (10)	0.0028 (7)	0.0021 (8)	0.0063 (8)
N2	0.0201 (10)	0.0292 (10)	0.0175 (9)	0.0060 (8)	0.0026 (8)	0.0064 (8)
N3	0.0176 (9)	0.0329 (11)	0.0173 (9)	0.0039 (7)	0.0045 (7)	0.0086 (8)
C1	0.0237 (12)	0.0291 (13)	0.0131 (11)	0.0043 (9)	0.0048 (9)	0.0041 (9)
C2	0.0198 (12)	0.0321 (13)	0.0284 (13)	0.0033 (9)	−0.0017 (10)	0.0077 (10)
C3	0.0151 (11)	0.0304 (13)	0.0225 (12)	0.0031 (9)	0.0002 (9)	0.0075 (9)
C4	0.0231 (12)	0.0371 (14)	0.0230 (12)	0.0088 (10)	0.0066 (10)	0.0097 (10)
C5	0.0267 (13)	0.0301 (14)	0.0300 (14)	0.0075 (10)	0.0017 (11)	0.0049 (10)
C6	0.0188 (12)	0.0337 (14)	0.0402 (15)	0.0008 (10)	−0.0007 (11)	0.0184 (12)
C7	0.0213 (12)	0.0544 (17)	0.0271 (13)	0.0045 (11)	0.0068 (10)	0.0164 (12)
C8	0.0231 (12)	0.0393 (14)	0.0227 (12)	0.0041 (10)	0.0040 (10)	0.0035 (10)
C9	0.0226 (12)	0.0241 (12)	0.0200 (11)	0.0021 (9)	0.0055 (9)	0.0041 (9)
C10	0.0195 (11)	0.0252 (12)	0.0182 (11)	0.0031 (9)	0.0037 (9)	0.0024 (9)
C11	0.0178 (11)	0.0318 (13)	0.0195 (11)	0.0010 (9)	0.0079 (9)	0.0031 (9)
C12	0.0225 (12)	0.0287 (13)	0.0200 (12)	0.0030 (9)	0.0088 (9)	0.0072 (9)
C13	0.0205 (11)	0.0255 (12)	0.0137 (11)	0.0026 (8)	0.0041 (9)	0.0011 (9)
C14	0.0165 (11)	0.0297 (13)	0.0235 (12)	−0.0007 (9)	0.0036 (9)	0.0074 (9)
C15	0.0224 (12)	0.0294 (13)	0.0228 (12)	−0.0009 (9)	0.0036 (10)	0.0090 (10)
C16	0.0260 (12)	0.0251 (12)	0.0207 (12)	0.0065 (9)	0.0049 (10)	0.0060 (9)
C17	0.0240 (12)	0.0287 (13)	0.0203 (12)	0.0056 (9)	0.0055 (9)	0.0081 (9)
C18	0.0170 (11)	0.0332 (13)	0.0194 (11)	0.0029 (9)	0.0027 (9)	0.0031 (9)
C19	0.0261 (13)	0.0457 (16)	0.0237 (13)	0.0038 (11)	0.0104 (10)	0.0013 (11)

### Geometric parameters (Å, º)

**Table:**

S1—C1	1.670 (2)	C8—H8	0.9500
S2—C1	1.751 (2)	C9—H9	0.9500
S2—C2	1.823 (2)	C9—C10	1.445 (3)
O1—H1	0.8400	C10—C11	1.398 (3)
O1—C17	1.429 (3)	C10—C15	1.400 (3)
N1—H1A	0.8800	C11—H11	0.9500
N1—N2	1.379 (3)	C11—C12	1.375 (3)
N1—C1	1.338 (3)	C12—H12	0.9500
N2—C9	1.286 (3)	C12—C13	1.420 (3)
N3—C13	1.371 (3)	C13—C14	1.411 (3)
N3—C16	1.459 (3)	C14—H14	0.9500
N3—C18	1.464 (3)	C14—C15	1.380 (3)
C2—H2A	0.9900	C15—H15	0.9500
C2—H2B	0.9900	C16—H16A	0.9900
C2—C3	1.498 (3)	C16—H16B	0.9900
C3—C4	1.390 (3)	C16—C17	1.509 (3)
C3—C8	1.395 (3)	C17—H17A	0.9900
C4—H4	0.9500	C17—H17B	0.9900
C4—C5	1.382 (3)	C18—H18A	0.9900
C5—H5	0.9500	C18—H18B	0.9900
C5—C6	1.379 (3)	C18—C19	1.521 (3)
C6—H6	0.9500	C19—H19A	0.9800
C6—C7	1.384 (4)	C19—H19B	0.9800
C7—H7	0.9500	C19—H19C	0.9800
C7—C8	1.379 (4)		
			
C1—S2—C2	101.28 (11)	C15—C10—C9	120.5 (2)
C17—O1—H1	109.5	C10—C11—H11	119.1
N2—N1—H1A	119.7	C12—C11—C10	121.8 (2)
C1—N1—H1A	119.7	C12—C11—H11	119.1
C1—N1—N2	120.63 (18)	C11—C12—H12	119.3
C9—N2—N1	114.97 (19)	C11—C12—C13	121.4 (2)
C13—N3—C16	123.26 (18)	C13—C12—H12	119.3
C13—N3—C18	121.00 (18)	N3—C13—C12	122.15 (19)
C16—N3—C18	115.70 (18)	N3—C13—C14	121.34 (19)
S1—C1—S2	124.96 (13)	C14—C13—C12	116.5 (2)
N1—C1—S1	120.51 (17)	C13—C14—H14	119.4
N1—C1—S2	114.53 (17)	C15—C14—C13	121.2 (2)
S2—C2—H2A	110.0	C15—C14—H14	119.4
S2—C2—H2B	110.0	C10—C15—H15	119.0
H2A—C2—H2B	108.4	C14—C15—C10	121.9 (2)
C3—C2—S2	108.51 (16)	C14—C15—H15	119.0
C3—C2—H2A	110.0	N3—C16—H16A	108.9
C3—C2—H2B	110.0	N3—C16—H16B	108.9
C4—C3—C2	120.6 (2)	N3—C16—C17	113.41 (19)
C4—C3—C8	118.8 (2)	H16A—C16—H16B	107.7
C8—C3—C2	120.6 (2)	C17—C16—H16A	108.9
C3—C4—H4	119.8	C17—C16—H16B	108.9
C5—C4—C3	120.4 (2)	O1—C17—C16	108.64 (18)
C5—C4—H4	119.8	O1—C17—H17A	110.0
C4—C5—H5	119.7	O1—C17—H17B	110.0
C6—C5—C4	120.6 (2)	C16—C17—H17A	110.0
C6—C5—H5	119.7	C16—C17—H17B	110.0
C5—C6—H6	120.3	H17A—C17—H17B	108.3
C5—C6—C7	119.4 (2)	N3—C18—H18A	109.0
C7—C6—H6	120.3	N3—C18—H18B	109.0
C6—C7—H7	119.8	N3—C18—C19	113.08 (19)
C8—C7—C6	120.4 (2)	H18A—C18—H18B	107.8
C8—C7—H7	119.8	C19—C18—H18A	109.0
C3—C8—H8	119.8	C19—C18—H18B	109.0
C7—C8—C3	120.4 (2)	C18—C19—H19A	109.5
C7—C8—H8	119.8	C18—C19—H19B	109.5
N2—C9—H9	119.0	C18—C19—H19C	109.5
N2—C9—C10	122.0 (2)	H19A—C19—H19B	109.5
C10—C9—H9	119.0	H19A—C19—H19C	109.5
C11—C10—C9	122.4 (2)	H19B—C19—H19C	109.5
C11—C10—C15	117.1 (2)		
			
S2—C2—C3—C4	86.2 (2)	C6—C7—C8—C3	−0.5 (4)
S2—C2—C3—C8	−95.4 (2)	C8—C3—C4—C5	−1.0 (3)
N1—N2—C9—C10	−175.48 (19)	C9—C10—C11—C12	174.2 (2)
N2—N1—C1—S1	−176.94 (15)	C9—C10—C15—C14	−176.0 (2)
N2—N1—C1—S2	2.7 (3)	C10—C11—C12—C13	1.0 (3)
N2—C9—C10—C11	16.2 (3)	C11—C10—C15—C14	0.8 (3)
N2—C9—C10—C15	−167.2 (2)	C11—C12—C13—N3	−176.9 (2)
N3—C13—C14—C15	175.2 (2)	C11—C12—C13—C14	2.2 (3)
N3—C16—C17—O1	−56.2 (2)	C12—C13—C14—C15	−3.9 (3)
C1—S2—C2—C3	175.21 (17)	C13—N3—C16—C17	98.0 (2)
C1—N1—N2—C9	−170.6 (2)	C13—N3—C18—C19	84.7 (3)
C2—S2—C1—S1	2.66 (17)	C13—C14—C15—C10	2.4 (4)
C2—S2—C1—N1	−176.95 (17)	C15—C10—C11—C12	−2.6 (3)
C2—C3—C4—C5	177.4 (2)	C16—N3—C13—C12	−0.4 (3)
C2—C3—C8—C7	−177.4 (2)	C16—N3—C13—C14	−179.4 (2)
C3—C4—C5—C6	0.5 (3)	C16—N3—C18—C19	−97.4 (2)
C4—C3—C8—C7	1.1 (3)	C18—N3—C13—C12	177.3 (2)
C4—C5—C6—C7	0.1 (4)	C18—N3—C13—C14	−1.7 (3)
C5—C6—C7—C8	−0.1 (3)	C18—N3—C16—C17	−79.8 (2)

### Hydrogen-bond geometry (Å, º)

**Table:**

*D*—H···*A*	*D*—H	H···*A*	*D*···*A*	*D*—H···*A*
N1—H1*A*···O1^i^	0.88	2.06	2.933 (3)	175
O1—H1···S1^ii^	0.84	2.36	3.1749 (19)	163

Symmetry codes: (i) −*x*+1, −*y*, −*z*+1; (ii) *x*−1, *y*, *z*−1.
